# The Profession of Apothecaries Degraded by the Abuses to Which Their Apprentices Are Subjected

**Published:** 1814-02

**Authors:** 


*For the Medical and Physical Journal.*

### *The Profession of Apothecaries degraded by the Abuses to which their Apprentices are subjected.*

Reformatores se reforment.

AMONG all the papers on Medical Reform which have been recently presented to the public, I do not recollect having seen a single paragraph that sufficiently exposes the abuses of Apprenticeships, which I am persuaded have done more towards degrading the profession of apothecary, than all other causes put together. Seeing then, that this branch of reformation has been so inadvertently or wilfully neglected, I shall with deference offer a few hints on the subject, which have been furnished by experience; hoping they will be received with some little indulgence, as they are suggested from no other motive than a wish to have truth appreciated, and vicious habits corrected.

The youthful mind is capable of almost any bent, and allurements will draw it from even favorite objects to those which duty or interest may render it necessary for it to be employed upon; so that few masters can reasonably attempt to cover their own negligence, by representing the fixed partiality of their pupil's inclination. It is the duty of a master to prepossess his apprentice in favor of his business, and instruct him in it; to make him sensible of its importance, and of the degree of responsibility attached to the professional character; to rivet his attention on it, allowing him to indulge in such other studies only as will ultimately contribute to the completion of his education and accomplishments. He ought to have an interest in his welfare, and show a tender solicitude for his promotion and improvement, recollecting that he is really a delegated parent, bound by law and reason to exert himself to the utmost in enforcing on his pupil good principles, and substantiating them by exemplary practices; in short, he ought to pay equal regard to the formation of the man, and the medical practitioner. Now this question obviously presents itself—*How are these*

*duties performed?*—Let an impartial public examine, and they will find that either indolence, jealousy, or avarice, has prevented a majority of masters from discharging them with punctuality, if at all; they will find that laudable curiosity and inquiry are suppressed, by cold, evasive, or indifferent answers, to questions proposed for the purpose of gaining information; they will find that youths ignorant and inexperienced, yet professing themselves fully competent, are daily substituted for age and reflection; and moreover, they may possibly discover, that the authors of these impostures have some of them, at least, no better intellectual endowments proportionally, than their ingenious representatives.

It may be urged that a great number of apprentices are careless, and show no desire to do their respective duties, or go about business in such a manner as evidently bespeaks their unwillingness. To which I answer, that such remarks (however just in many cases) can never apply to the young apprentice, who is always on the alert to please his master, and procure his esteem; who enters the business eager to improve, and gain a knowledge of its principles. They are not admissible till after he has discovered that the modern practice of medicine is frequently a trade, conducted by certain established rules, attainable only by practice; till he has found himself decoyed into an engagement which will retard his future progress in scientific pursuits, and confine him to a counter for four, five, or six years, the most valuable in his life, all to worse than no purpose; till he is disgusted with the taciturnity of his master, whom he had reason to expect would be a friend and preceptor, to correct and fix his wavering opinions. And if these circumstances are not sufficient to palliate, if not justify, the remissness of a young man, I must confess I have hitherto had but very incorrect notions of the principles of moral equity.

At the beginning of an apprenticeship, that plan of behaviour should be adopted which is most consistent with the present circumstances and future prospects of each party. If a master expects attention and respect from his pupil, he must, by setting an example, secure such an imitation as accords with his wishes: he cannot reasonably suppose, that unconditional obedience is always subsequent to supercilious command, or attempt to frighten any one into an approval of his ridiculous notions, however supported by superiority or sanctioned by experience. No master can, without proper instruction and proper conduct, gain the good will and wishes of his apprentice; and very few with them can miss the attainment of that desirable object. Do we expect to find in the young and inexperienced, or in the aged, stability of deportment? Will an abject fear of consequences, or a grateful remembrance of past kindness, tend most to conciliate the affections, and of course command the good offices of young men? Did I not avoid personality, I could adduce instances of youths, who, after serving a five or six years' apprenticeship, had acquired such liberal notions of medical practice, as to recommend invariably Inf. Rosæ in simple fever; of others, who totally discarding that elegant medicine, as regularly would propose Mist. Sal. cum Bol. Antim. Could these worthies act from a conviction in their own minds of the superiority of their favorite specifics, or did they want an enlargement of intellect to comprehend that though they had seldom, if ever, witnessed the exhibition of different medicines, there were others equally efficacious? I presume, their separate masses of intelligence were such as their own limited practice, or empirical habits afforded. What sandy foundations on which to erect future professors of medicine, on whose abilities hundreds may depend for the preservation of their health or lives! I cannot bring myself to suppose that there is one individual who would not wish for a perfect renovation, seeing that, in a great measure, his character and respectability increase or diminish with his professional celebrity. Admitting, then, the preceding statements to be true, (and every day's experience supplies incontrovertible evidence of their truth,) there is no method of permanently improving the profession, but by a well-regulated education; that is the basis on which the superstructure of experience must stand. A man, accustomed from his first attendance at the bed of sickness, to judge with deliberation, and observe minutely the most intricate and often delusive forms of diseases, will profit more by one than an empiric will by twenty years experience. Then how necessary it is that apprentices should be taught to begin and proceed upon rational principles, in order that they might acquire a habit of thinking for themselves, and of "analyzing disease."

I think it would not injure the reputation of a master, if he were to take his apprentice, in all cases where it was admissible, to the bedside of the patient, that he might be present at the examination; and afterwards explain to him his motives for giving medicines, the effects he expected them to produce, the state of the disease, its manner of injuring the system, and its probable consequences: it would rather rather induce the apprentice to love and respect his master, and give him an interest in the practice, which would keep alive his curiosity, and compel his attention. But it would require more familiarity or condescension than many men are capable of; their pride is alarmed at the idea, their honor revolts, they cannot think of being seen in friendly conversation with their apprentice, it would sink them too much, if any thing can sink them more, in the estimation of the thinking part of mankind, than their present shameful negligence. Yet in all honest minds, this false pride and mistaken honor will yield to the reflection, that on their present conduct the future usefulness and respectability of the rising generation of practitioners depend; for surely no one is so depraved as to wish that his profession should fall into contempt and ignominy. I would ask every master, whether he would rather that his apprentice should pass through a life of honorable celebrity or obscure meanness? I would appeal to the dictates of his conscience for an answer; and propose that he should adopt such plans of proceeding as are most consistent with his own credit and advantage.

But to show in a clearer light that the present practices are inimical to the cultivation of medical science among the industrious, as well as the careless and inconsiderate, let us suppose two young men bound to an *eminent* apothecary, who, (like many great modern reformists,) considers them as his servants, and expects no more trouble on their account; we will admit one an active and penetrating genius, the other an easy unconcerned observer of things, after the first impulses of his curiosity are gratified. Now, it is evident that the one, anxious for information, would continually refer to books for that which no living instructor would supply; he would gain a mass of indigested crude facts, unarranged by judgment, or unregulated by reason; he would be always at a loss in practice for a *rational theory* and *established principles* to direct him in the use of his already acquired knowledge, in spite of which, however extensive it might be, he would be perfectly impotent: and as these disadvantages increase with his diligence, it would be good policy in him to neglect his medical studies entirely, and engage with such as his mind enabled him to advance in, or his taste selected as most agreeable. The other would be quite different; born with a superior capacity, and formed for enterprize, he would attentively acquire a few dogmatical facts, upon which he would erect his professional importance, (which he undoubtedly would have no mean opinion nion of;) he would conceive himself perfectly qualified to prescribe for his *devoted victims*, and to "*wrestle with Death*;" supported by a train of empirical notions, his conscience well barricaded against the innovations of humanity. Thus both would be uninformed, in almost an equal degree; only, one would be sensible of his ignorance, and the difficulties he had labored under, and consequently well prepared for improving in a more eligible situation; while the other would be deprived of that necessary state of mind for receiving instruction, supposing himself at the acme of perfection, and wanting nothing but opportunities to display his powers, which might raise him to the highest pinnacle of fame.

That country apprenticeships are a very inefficient mode of education, their most sanguine advocates cannot deny: that their present practice in most cases is not infamous, I am convinced neither argument nor sophistry can prove. That ignorance in the profession originates here, is evident; and however the Committee of Apothecaries may exert themselves to bring about a reform, unless that reform begins to operate in the education of every apprentice, I confidently predict, that neither the profession nor the public will be materially benefited; and if it does, little more will be necessary. A certain number of men may be chartered, and gain useful privileges; but whether they will extend beyond the limits of their synod, so as to be of real benefit to society, I think we may reasonably doubt; for notwithstanding all the specious reforms that can be obtained, one truth will still be evident, viz. *that no body of men as a profession can excel, whose mode of education is radically bad.** 

Now, there are few who are not conscious of the existence of the abuses pointed out, but it remains to be said how they can be remedied. It is not without some diffidence that I make the subsequent remarks, being aware that it is easier to detect and expose corruption, than to suggest proper plans to be adopted for its amendment. However, I shall give such opinions as have occurred to me, on deliberate reflection; and here I cannot but express my regret that a respected and useful profession should require public notification of their negligencies in the education of their youth, a

* Might not a petition from apprentices be presented to parliament, (briefly praying that they might no longer be tricked out of their time and money by this unhappy system, but have their griefs redressed, and oppressions removed,) with as much propriety and more justice than those of the apothecaries can boast?

duty duty of the highest importance both to themselves and to mankind. In the name of humanity, whence originate these direful abuses, which are a just stigma to the age, as unaccountable as they are general, as injurious as they are enormous; which destroy the buds of genius, blast the shoots of imagination, retard the indefatigable, encourage the indolent, murder hope, and confirm ignorance, prejudice, and stupidity? May they be banished from amongst us, that we may no longer suffer under the weight of their oppression, and that the recollection of them may be obliterated for ever. The best way (as circumstances are) for all parties to be brought to a proper discharge of their duties, will be to reflect that they are bound to them not only legally, but morally, and that the obligation does not cease with them, but extends to every branch of civilised society; and though a master and his apprentice may agree between themselves, or coincide in opinion on the business, the public are not to be satisfied with their ignorance, or gulled by their specious pretensions to talent. Even this consideration, that in the tuition of a young man, there is confided to the preceptor the health of numbers, in a manner, whom he never will know, but who may come under the care of the future practitioner, whose principles he is bound to inculcate,—is sufficient to convince every unprejudiced and liberal man of the importance of this duty, and stimulate him to a full discharge of it. Every day must bring unnumbered perplexing difficulties, against which a good master will fortify the mind of his pupil, knowing that the public judge of a medical man's abilities by his promptness in unforeseen exigencies, and success in general practice, on which depend his future dignity or degradation in society. If a master should have a refractory pupil, or a pupil an obstinate master, let a necessary disunion be effected as soon as possible, that neither party may suffer obviable injury or deprivations. Should apprenticeships continue to be conducted in the same way as at present, I feel no hesitation in recommending to parents a discontinuance on their parts in supporting them, but would propose *that a youth, from the first, should be placed under the care of eminent professors, in one of the first medical schools, and continued there through the whole course of his studies, that they might be conducted in a manner at once agreeable and advantageous, complete and rational.*

To all well wishers to the profession, I beg leave to recommend this reflection: *if the bill at present in agitation should pass into a law, these practices complained of will be partly sanctioned by legal authority, and there is no clause in*

 *its*

*its present form that provides a remedy for, or even so much as mentions them.* What then can be expected but their continuance and increase? It is very proper that the legislature should be informed of these *radical evils,* that they may not unknowingly perpetuate them, by enacting that a five years apprenticeship shall be served previous to hospital attendance; for of these five years, four will be murdered time, even allowing that the apprentice shall see constant practice, for, as is before observed, he cannot be able to improve by it. All that an apprentice can learn during his servitude, amounts to nothing compared with what a man in practice should know: he may study the materia medica, and the first lines of the practice of physic and surgery, together with a little midwifery; he may learn to extract teeth, bleed, exhibit an enema, dress a simple wound, pull at a fractured limb, and look grave, *but no more.* Now these, sir, are incontrovertible facts, these are the incalculable advantages of a five years compulsory servitude; and yet the committee for regulating practice have entirely overlooked these pernicious principles, and conveniently forgotten to expatiate on the *evils* inseparably united with this injurious system. When I think on the tender sensations of gratitude which rise in the mind of a well-disposed pupil on the remembrance of his tutor's kind solicitude and unremitted exertions for his welfare, on the pleasing reflections that man must have who has been the mean of disseminating virtue and knowledge through a rising and grateful generation, I am shocked to observe these tasks so palpably, so wilfully neglected. I examine for a cause, and find, painful reflection! that intellectual enjoyments are superseded by avarice; that momentary gratifications are preferred to lasting pleasures; and that men (as a writer of judgement observes) "not being able to lift their eyes above this world, think nothing worth their care but raking together the dross which it affords, each striving, like the toad, who shall die with most earth in his paws." But there are individuals whose liberal generosity is equalled by nothing but their constant exertions towards benefiting their profession: to such I look with gratitude; of such, who are solitary instances of independent virtue, and unbiassed goodness, I speak with respect and reverence: may they continue to show their admirable example, that the kind spirit which animates them may diffuse itself through the rest of their brethren, and that one principle of perfect amity may actuate the whole!

*Yorkshire,* JUVENIS.

